# Economic Cost and Health Care Workforce Effects of School Closures in the U.S.

**DOI:** 10.1371/currents.RRN1051

**Published:** 2009-10-05

**Authors:** Howard Lempel, Joshua M Epstein, Ross A Hammond

**Affiliations:** ^*^Economic Studies, The Brookings Institution and ^†^The Brookings Institution

## Abstract

School closure is an important component of U.S. pandemic flu mitigation strategy, but has important costs. We give estimates of both the direct economic and health care impacts for school closure durations of 2, 4, 6, and 12 weeks under a range of assumptions. We find that closing all schools in the U.S. for four weeks could cost between $10 and $47 billion dollars (0.1-0.3% of GDP) and lead to a reduction of 6% to 19% in key health care personnel.

## 1) Introduction

    School closures are an important and controversial part of the U.S. federal government’s Community Strategy for Pandemic Influenza Mitigation in the United States [1].  Closing schools would reduce social contacts and suppress transmission.  A recent literature review concludes that the direct epidemiological benefits of such a strategy are uncertain and depend on the specifics of implementation, but could include a modest (~15%) reduction in total cases and a large (~40%) reduction in peak attack rates [2].  

    Controlling the peak attack rate will be crucial to prevent the U.S. healthcare system’s surge capacity from being overwhelmed.  In its model scenario, the President’s Council of Advisors on Science and Technology (PCAST) finds that flu cases may demand 50 to 100 percent of the total intensive care unit (ICU) capacity in the United States.  This is a major threat to a system that operates at 80 percent of capacity during normal times [3].  

    Closing schools is controversial because the epidemiological benefits come with associated costs.  With their children out of school, many parents will stay home from work.  This absenteeism will lead to significant economic costs.  Compounding the problem, some absentees will be health care workers.  The most pronounced benefit of school closure is to alleviate pressure on the health care system.  But if health care absenteeism is high, the system’s capacity could be reduced when the virus is most prevalent and the demand for health care services is highest.   

    PCAST emphasizes the lack of research on the magnitude of these formidable costs: “Although evidence-based estimates of such costs are difficult to make and inherently imprecise, they can help to advance the rationality of the debates . . .”  To that end, this paper includes the first detailed estimate of two of these costs in the United States. We estimate the economic cost of school closure and its impact on the health care system.  In addition to providing the first detailed estimate using U.S. data, we enrich the existing international literature in three ways.  First, we use a more comprehensive method to identify adults who stay home to provide care.  Second, when calculating the value of work missed by caretakers, our data allows us to use the caretakers’ actual wages for all caretakers who are not self-employed.  Third, recent survey data enable a precise estimate of the proportion of the workforce that is able to work from home and the makeup of this segment of the workforce.  We find that closing all schools in the U.S. for four weeks could cost between $10 and $47 billion dollars (0.1-0.3% of GDP) and lead to a reduction of 6% to 19% in key health care personnel.

## 2) The Simulated Policy

    We estimate the effect of proactively closing all schools and formal daycare centers in the United States.  This is the same policy analyzed in two previous studies of the costs of school closure [4] [5].  Some researchers have proposed “reactive closure” or “class dismissal” instead of “proactive closure,” the policy we consider.  In reactive closure schools are closed once many children and/or staff have fallen ill.  In class dismissal schools remain open, but most children stay home [2].  We report estimates of the per child costs of closing school so our results can be extended to these other cases.

    The CDC’s Community Strategy for Pandemic Influenza Mitigation contains recommendations for the length schools should remained closed for pandemics of various severities.  In the event of a Category 2 or 3 pandemic,the CDC recommends that closure of up to 4 weeks be considered.  In the event of a Category 4 or 5 pandemic, closure of up to 12 weeks is recommended [1].  Our simulations vary the length of closure from two to twelve weeks to capture the entire range of CDC recommendations. 

## 3) Previous Literature

    In the most comprehensive existing study, Sadique et. al. (2008) find that closing all schools for four weeks in the United Kingdom would cost between 0.1% and 0.4% of GDP.  Sadique et. al. estimate the costs of school closure in the U.K. by identifying workers likely to be primary caretakers for children, adding up estimates of their wages, and adjusting for workers who cope with a closure by using informal child care, working from home, or making up work in the future.  In addition to applying their analysis to the United States, our estimates build upon Sadique et. al.’s methodology in three ways [4].

    First, to identify adults who are likely to stay home to care for children, Sadique et. al. assume that children are cared for by household heads or their spouses.  However, we believe that an adult’s relationship to a child is a better predictor than head-of-household status, which describes legal housing relationships but not child care relationships.  We are also able to adjust for the presence in the household of adults, such as fathers, siblings, relatives, or housemates, who are not working or working part-time and could provide costless care for children.^1^

    Second, when calculating the value of work missed by caretakers, we use the caretakers’ actual weekly wages for all caretakers who are not self-employed.  Sadique et. al. assume that caretakers make the average weekly wage for workers of their industry and gender, that part-time work is equally prevalent within each industry/gender cell, and that the part-time workers work half as many hours at the same hourly rate as full-time workers.  These assumptions do not hold in our data.

    Third, we use survey data to precisely determine how often workers will be able to work from home if they are caring for their children.  Sadique et. al. do not have data on ability to work from home and therefore use data on access to broadband as a proxy.  Unfortunately in many industries, for example most of the service sector, working from home is impossible regardless of any access to broadband.  Our data is also broken out by household income, allowing us to adjust for the fact that workers in high-income households are more than three times as likely to be able to work from home as workers in low-income households [6].

    In a paper simulating the net economic effects of a vast array of mitigation strategies, Sander, et. al. provide the best existing estimate of the cost of closing all schools in the *United States* [5].  They find that the net cost of closing schools in the United States for 26 weeks is $2.72 million per 1,000 persons.  They reasonably assume that 2.5 days of work are missed per week in each household with a child.  However, they make no effort to adjust for the difference in earnings between adults who are likely to care for children and other workers.

## 4) Methods

    To estimate the costs associated with school closure, we first use the Current Population Survey (CPS), a large, household-based labor force survey in the United States that is representative of the civilian non-institutionalized population, to identify households where an adult is likely to miss work to provide child care.  Next, we select adults that are most likely to miss work based on their gender and relationship to children in the household.  Finally, we use data on absentees’ earnings and industry to value the effect of their absence on the economy and the health care system.  In our low estimate, we also adjust these costs downwards to account for the use of informal child care, some workers’ ability to work from home, and an elasticity of output with respect to hours that is less than unity.  In this section, we discuss each of these steps in turn. 

### **A) The Data**

    Most of our data are extracted from the 2008 CPS.  The CPS is a monthly labor force survey that asks respondents detailed questions about their labor force involvement and relationships to other members of the household.  Data on sex, household relationships, and labor force status allow us to identify likely absentees.  Data on hours worked in the previous week allow us to estimate the amount of work missed and data on the previous week’s earnings aid our valuation of that work.

    We restrict our sample to members of the CPS’s outgoing rotation groups (ORGs).  CPS respondents are surveyed eight times.  When a respondent enters the CPS, she is surveyed each month for four straight months.  For the next eight months, she is removed from the sample.  She is then interviewed each month for four more months to finish out her time in the survey.  Respondents in their fourth or eighth month in the survey are said to be a part of the outgoing rotation groups because they will not be surveyed again in the following month.  To minimize respondent burden, the CPS only asks questions on earnings and hours of respondents who are in ORGs.  We merge data from each month’s ORGs, creating a large sample of 405,211 observations including 191,841 workers.

    Unfortunately, even ORG members are not asked about their earnings if they are self-employed.  For these workers, we turn to the 2008 Annual Social and Economic Supplement (ASEC), which has data on workers’ total earnings and weeks worked in 2007.     

    Data on the use of informal child care comes from the eighth wave of the Census’s 2004 Survey of Income and Program Participation (SIPP).  The SIPP is a nationally representative panel survey designed by the Census to provide information on the principal determinants of income and program participation in the United States.  Respondents in the late spring and summer of 2006 were asked about childcare arrangements of children under age 15 whose guardians are working or in school.     

    Data on the ability to work from home comes from the Harvard School of Public Health Project on the Public and Biological Security’s *Pandemic Influenza Survey* [6].  The survey, administered in the Fall of 2006, included a nationally representative survey of 1,697 adults age 18 and over with an over-sample of adults in households with children.  The surveys author’s have published data on the ability to work from home for one month during a pandemic by household income level.  Respondents who would be able to work from home were also asked whether they could work from home while caring for their children [7].

### **B) Valuing Lost Production**

    If an adult stays home from work to care for a child, the output she would otherwise have produced is lost.  The “human capital method” (HCM) is the most common way to value this lost output.  Neoclassical economic theory implies that under perfect competition workers’ marginal compensation is equal to their marginal revenue product.  The HCM assumes that perfect competition holds, so the cost of a week’s lost work is equal to a week of compensation [8]. ^2^     Koopmanschap et. al. argue that the HCM overestimates the costs of absenteeism by neglecting the ways firms can cope.  As long as there is a reserve of unemployed labor, workers absent for long periods of time will be replaced.  Therefore, the true cost of absenteeism is not equal to the worker’s compensation over the length of her absence, but rather to the total of her compensation during the time it takes to replace her, and the costs associated with finding and training her replacement [9].  Koopmanschap et. al. dub this the “friction cost method” (FCM) for valuing the indirect cost of disease, absenteeism.

    Sadique et. al. point out that the HCM and the FCM should produce similar estimates of the costs of school closure because schools are not expected to close for so long that replacement workers will be hired and trained.  Therefore, like Sadique et. al., we use the HCM to value the costs of absenteeism due to school closures.  Unfortunately, we know of no large household-based datasets with reliable data on fringe benefits, so we use earnings as a proxy for compensation [4].^3^  We must also confront a second data issue.  Our main dataset, the CPS ORGs, is missing data on earnings among self-employed workers.  We impute these earnings using data on annual earnings and weeks worked among self-employed persons in the 2008 CPS ASEC.^4^

### **C) Who Stays Home?**

    Our baseline estimate assumes that, in the event of a school closure, one adult (age 16+) member of every household with at least one child (age <16) must be at home to provide care at all times.  This means that no work will be missed in any household containing an adult who did not work in the week prior to being surveyed.  In households where every adult works, one employed adult will miss work and provide care.

    Next, we must predict *which* household member will stay home.^5^^^ In our baseline estimate, we make two assumptions: 1) Adults who are closely related to a child are more likely to care for her than other adults; 2) If male and female adults are equally closely related to a child, a female stays home.  Specifically, we break adults down into six categories: 1) Mothers of children in the household; 2) Fathers of children in the household; 3) Other female relatives of children in the household; 4) Other male relatives of children in the household; 5) Other females; and 6) Other males.  If the mother of any child in the household is present, then she is assumed to stay home and care for all of the household’s children in the event of school closure.  If more than one child’s mother is present (e.g. in a multi-family household), then one mother is randomly selected to stay home from work.  If no mothers are present, then an adult from our 2^nd^ category (i.e. a father) is selected to stay home.  If no child’s father is present, then an adult from category 3 is chosen, and so on.^6^ ^7^  In our high-cost scenario, we assume that households choose adults to care for children without regard to gender.^8^

    Fortunately, not all caretakers will have to miss all their hours.  If someone in the household works part-time then the care taker only misses as many hours per week as are worked by the adults who work the least.  As an example, consider a household with one child; suppose her mother works 40 hours per week, her uncle works 20 hours per week, and her aunt works 5 hours per week.  We assume that the aunt can care for the child when she is not at work, so just 5 hours of work per week will be missed by the child’s care taker.^9^

    We believe this approach yields a reasonable baseline for the level of absenteeism the U.S. will face in the event of an epidemic.  Multiple children should not be cared for together because this would negate the epidemiological benefits of school closure.  This means that care takers will be scarce in the event of an epidemic and few households may be able to find babysitters or neighbors to care for their children.  Survey evidence confirms the plausibility of our baseline.  In a recent Harvard School of Public Health survey, 51% of respondents in households with children in school or daycare reported that it was likely that an adult in their household would have to miss work if schools were closed for two weeks [10].  By comparison, in our baseline estimate an adult misses some work in 42% of households with children under 18.

    Following Sadique et. al., in our low estimate, we allow households to avoid missed work by turning to informal child care or by working from home.  Many households with children and without stay-at-home adults already use informal child care.  Children are often cared for by grandparents, other relatives, family or family daycare providers^10^while the adults in their households work.  Other children care for themselves.  Because children in these arrangements are likely to encounter less social contact than at school, many households may elect to use informal care to allow employed adults to keep working if schools close.

    According to the 2004 SIPP, during normal times, at least one child regularly^11^cares for herself, or is cared for by a relative other than her parents^12^ ^,^ a baby sitter, some other adult, or a family day care provider in 57% of households with children under the age of 15 and where every adult works.  We assume that these households will expand their use of informal care instead of having an adult miss work and that this expanded use of informal care does not cause any other adults to miss work.^ 13^^^ Therefore, in the low cost estimate, we multiply the lost output of all workers by 0.43.^ 14^

    Other workers will cope with an epidemic by working from home.  In a 2006 HSPH survey, 25% of employed respondents reported that they would be able to work from home for one month while caring for children in the case of a pandemic.  This included 11% of persons in households with income under $25,000, 18% in households making $25,000-$49,999, 23% in households making $50,000-$74,999, and 38% of persons in households or more $75,000 in income [6 7].^ 15^ ^16^ We multiply lost output in these households by 0.89, 0.82, 0.77, and 0.62 respectively to account for workers who work from home.^ 17^

    Overall, in the low cost scenario, a worker misses some work in 14% of households with kids under the age of 18.  We believe this leads to a very conservative estimate of the costs of school closure.

### **D) Calculating Economic Costs**

    In our baseline estimate, the total economic cost of absenteeism due to school closure is calculated as the sum of the weekly earnings for all caretakers multiplied by the length of the school closure.  However, output does not necessarily decrease proportionately with hours.  If a worker misses a week of work, her colleagues might pick up the slack or she might work harder the next week to catch up, so production might decline by less than 2%.  On the other hand, if her work is team-based or extremely time-sensitive, then her absence might have a disproportionately large effect on the firm’s output.  Saqidue, et. al. found that  the best estimate of the elasticity of output with respect to hours of labor^ 18^ is 0.8.  In other words, 20% of missed production will be made up by the worker herself or her colleagues.  Therefore, in the low-cost estimate, all lost output is multiplied by 0.8.

### **E) Estimating Absenteeism in the Healthcare Sector**

    The scenarios that would induce high *economic *costs during a school closure are not the same scenarios that would induce the most stress on the healthcare system.  78% of healthcare workers are women, so absenteeism in the healthcare sector would be *lower* if more men were to care for children, as in our high-economic-cost scenario.  Moreover, few if any healthcare workers can be effective working from home.  We therefore report two estimates of absenteeism in the healthcare sector.  Our baseline estimate assumes the same scenario as in our baseline economic estimate.  Our low-cost estimate of the impact of school closure on health care absenteeism assumes that households 1) choose caretakers for children without regard to gender; 2) expand their use of informal care instead of missing work if they already have an informal care system in place.  As in our baseline scenario, we assume that workers are unable to work from home and that the elasticity of output with respect to labor hours is one.  Healthcare provision during a flu epidemic is time-sensitive (so workers cannot make up missed work when they return) and the healthcare system will be overburdened (so coworkers will not pick up slack).   We therefore believe it is unreasonable to assume that healthcare provision will decline less than proportionately with hours of work missed. 

## 5) Results

### **A) Overall Absenteeism**

    In the United States, 23% of all civilian workers in the United States live in households with a child under 16 and no stay-at-home adults.  Under our baseline scenario, 52% of these workers will miss some work to care for children, and 10% of all labor hours in the civilian U.S. economy will be lost during the period that schools are closed.  If many workers are able to cope with closure by turning to informal childcare and working from home, as in our low-cost scenario, 3% of all labor-hours in the civilian U.S. economy will be lost.

    In our baseline scenario, 95% of absentees are female.  In our high cost scenario, where households choose caretakers without regard to gender, a majority (59%) of caretakers are still female because single-mother households are much more common in the United States than single-father households. 

### **B) Economic Costs**

    Table 1 and Chart 1 present our estimates of the economic costs the U.S. would face if schools were closed for different lengths of time.   The federal government’s most recent community strategy for pandemic influenza mitigation recommends adapting community responses based on a pandemic severity index, which varies from category 1 (similar to the seasonal influenza) to category 5 (similar to the 1918 pandemic).^ 19^^^ The plan calls for communities to consider closing schools for four weeks or less if the index hits two or three, and recommends that they close schools for up to twelve weeks if a pandemic is even more severe.  We focus on the costs of closing schools for four weeks, approximately at the midpoint of potential closure lengths.

    According to our baseline estimate, closing schools for four weeks would reduce U.S. GDP by about $43 billion dollars or 0.3% of last year’s total GDP.  Because men earn more on average than women, the costs will be even greater if households decide to distribute caretaking responsibilities without regard to gender.  In our high cost scenario, $47 billion dollars is lost.  On the other hand, work-from-home, informal child care, and other coping strategies could substantially reduce these costs.  A one month long school closure decreases U.S. GDP by $10 billion dollars in our low cost estimate.

    Recent newspaper reports suggest that the Obama Administration is moving away from a preemptive school closure strategy toward a policy of more targeted interventions [11].  Table 2 provides estimates of the per student^ 20 ^weekly cost of closing schools.  We divide the weekly economic cost of closing schools throughout the U.S. by the total number of students in the U.S. and find that closing schools for four weeks would cost between $140 and $630 per student.  This allows us to estimate the costs of localized school closure, which could take place in areas that are especially hard-hit.^ 21^ For example, there are 115,000 students in Washington, DC, so closing all DC schools for one week might cost between $4 million and $18 million dollars.  Estimates of the cost of school closure per student also facilitate comparison of the cost of closing schools with the cost of other mitigation strategies.

### **C) Impact on Households**

    These aggregate macroeconomic costs will affect different households in very different ways.  Because their earnings tend to be greatest, absenteeism among high-income households contributes the most to overall costs.  However, high-income households are likely to be most able to cope with lost income, so this measure may not accurately reflect the likely distribution of hardships if schools are to close.

    Specifically, hardships may be minimized in households with many earners.  While one adult misses work to provide care, other workers will continue to bring in earnings and support the household.  However, households with just one adult may have to cope without any earnings at all while that adult is absent from work.  In fact, 20% of households with absentees contain just one adult and will be left without earnings if she is forced to miss work.  Most of these households are headed by single parents, who are disproportionately likely to have low- or moderate-income.  Specifically 43% of the households expected to lose all of their earnings during a school closure are already in the bottom quintile of household income.^ 22^ When considering school closure, policymakers must consider the disparate impact such a policy is likely to have on single parents and low-income households.  They should consider strengthening safety nets to minimize the financial distress closing schools causes for these workers.

### **D) Educational Costs**

    This paper specifically focuses on estimating the cost of workplace absenteeism brought about by closing schools.  However, another important cost of school closure is student learning loss.  In this section, we briefly address this cost.

    One could value learning loss using the human capital method, adding up the earnings of all persons in the education sector in the same way that we valued the costs of absenteeism.  This approach assumes that the total earnings of all educators in the United States are equal to the value of all formal education.  We find that the total earnings of all persons who work in elementary or secondary schools are $7.3 billion per week.  We can improve this estimate by accounting for two additional facts.  First, there is some overlap between these costs and the economic costs estimated previously, because some workers in the educational sector were already assumed to stay home to care for their children.  Second, if we assume that workers in the educational sector do not work at all during school closure, then these educators will be free to stay home and care for children of their own, and other members of their households who otherwise would have stayed home to provide care will be able to attend work.  After accounting for these factors, we find that adding the cost of lost learning increases the weekly cost of school closure by $6.1 billion dollars.

    The U.S. educational sector is not, however, a world of perfect competition.  It would be quite optimistic to assume that the political processes that set most educators’ pay leads compensation in that sector to be equal to education's social value, especially because of the high positive externalities involved in education.  Therefore, we turn to the literature on summer learning loss for a second estimate of the educational costs of school closure

    Cooper et. al. have carried out a meta-analytic review of the research on summer vacation’s effect on achievement test scores [12].  They find that on average students lose about one month of learning over a summer break.  This average hides substantial variability by socioeconomic status.  While the math scores of students from lower- and middle-income families backslide equally, lower class students’ reading scores decline over the summer, while middle class students’ scores increase.  If school closure during an epidemic has a similar effect on student achievement then it could have adverse effects on educational inequality.  Worse yet, researchers have hypothesized that differences in backsliding are caused by enrichment activities available to middle-class students over the summer.  If schools were suddenly closed due to a flu epidemic, these opportunities might not be available and middle-class students may face backslides similar to students from low-income families. 

### **E) Stress on the Health Care Sector**

    A main goal of closing schools is to delay and lower peak attack rates in order to avoid surpassing the healthcare system’s surge capacity [2].  The health care workforce is central to the mitigation strategy.  If closing schools leads healthcare workers who would be treating or vaccinating patients to stay home from work and watch their kids, then the benefits of school closure may be undermined by the absenteeism it generates.  Closing schools during the epidemic could lead the supply of health care workers to be at its minimum precisely when they are most needed.

    The appropriate unit in which to measure this effect is the percent of work-*hours* foregone by healthcare workers providing childcare.  We emphasize that we estimate *only* the rate of absenteeism due to school closure.  During an epidemic, the rate of absenteeism will already be high, as many workers are expected to miss work due to illness or fear of the same.  Citing U.K. data, Sadique et. al. expect that 15% of the healthcare workforce would be absent during an epidemic *in the absence of school closure* [4].

    In our baseline estimate, 18% of all hours in the healthcare sector are lost.  However, many healthcare workers will not be involved in influenza mitigation.  It is unlikely that workers in the offices of dentists, chiropractors, or optometrists will aid in flu mitigation, so we are most concerned about absenteeism in other industries^ 23 ^.  In our low-cost scenario, 6% of work hours will be lost due to school closure in these industries, while 18% of hours will be lost in our baseline scenario.

    These flu-relevant sub-industries include audiologists and physical therapists, radiation therapists and speech-language pathologists.  These workers will not be involved in flu treatment.  Therefore, to add further precision to our estimate of stress induced on the healthcare system, we estimate hours lost in relevant industries among workers in relevant occupations.^ 24^^^ We find similar results: in the low-cost estimate, 6% of work hours in relevant industries *and* occupations are expected to be lost, while 19% of work hours are lost in the baseline estimate.     Overall, we find that closing schools would risk inducing a substantial increase in health care absenteeism just as demand for healthcare hits its peak.  Policymakers should consider this cost when weighing the epidemiological benefits of closing schools.

## 6) Conclusion

    We find that closing schools in the United States for four weeks would reduce U.S. GDP by between $10 and $47 billion dollars, a cost equivalent to 0.1% to 0.3% of 2008 U.S. GDP.  In addition, such a policy could lead to the absence of 6-19% of relevant healthcare personnel just when these workers are most needed, when incidence is high.     Our study builds on the work of Sadique et. al. who use data from the United Kingdom to estimate the costs of absenteeism due to school closure.  In addition to publishing new detailed estimates using data from the United States, we add precision to previous methods by better identifying caretakers, their actual salaries, and their ability to work from home.     Opportunities remain for future research to more precisely estimate the relevant costs.  More work must be done to identify households where someone will stay home from work and to identify the most likely child care providers in households with multiple adults.  Ideally, better data will allow researchers to model households’ decisions taking into account the age of children who are present and each worker’s salary, fringe benefits^ 25 ^, and ability to work from home.  Last spring’s school closures present an excellent opportunity to collect such data.  Similarly, more data on the actual use of informal child care and working-from-home during school closures would improve the precision of cost estimates.  Accounting for fringe benefits might substantially increase the estimates presented here, which are conservative.^ 26^

    Another contribution would be to embed detailed estimates of the cost of school closure into a more general epidemiological model.  In an epidemic, some workers would miss work due to fear, their own illness, or their children’s illness, even if schools remained open.  A full-scale epidemiological model could estimate the number of caretakers who would have been absent even if schools remained open.

    Our paper does not attempt to account for any multiplier effect brought about by a multi-billion dollar decline in economic output.  The decrease in aggregate supply might lead to inflation.  If workers who stay home to provide child care are fired or must take unpaid leave, the collapse in aggregate demand could lead to further reductions in output.  These effects could be captured if the policy simulation were embedded in a macroeconomic model.

    Finally, more research is needed on the structure of health care providers’ production functions and precisely which workers will be involved in flu treatment.  For example, some healthcare workers might be easy to substitute for, while the absence of others might severely degrade the healthcare system.  The distribution of absences might also matter.  If a 19% absenteeism rate due to school closure is evenly distributed throughout the healthcare system, providers might be able to adapt.  But, if a hospital is missing *all* its nurses, treatment might be more severely impeded.  A larger dataset would allow a breakdown of absenteeism by detailed occupation within relevant sectors of the healthcare industry.  It is not clear what level of absenteeism at the epidemic peak would undermine containment: the delivery of vaccine, the treatment of cases, or the implementation of other mitigation measures.  But this is clearly a central question raised by this research.

    If the delivery system degrades “ungracefully,” suddenly becoming dysfunctional at a threshold, we need to know where that threshold is and avoid it. We have shown that closing schools at the height of an epidemic could bring about the loss of up to 19% of work hours in the healthcare sector.  If other causes of absenteeism are included, personnel loss may be as high as 34%.  Policymakers must factor this cost into any cost-benefit analysis of school closure.  If schools do close, possibilities would include special arrangements for health care workers’ children (e.g. priority vaccination or non-parental supervision which could be provided at low-cost by sidelined workers from the educational sector).  We venture no conclusions on this topic, but believe it warrants intensive analysis. 

## Acknowledgements

The authors thank Julia Chelen for her assistance.

## Funding Information 

This work was supported by:  The Models of Infectious Disease Agent Study (MIDAS), under Award Number U01GM070708 from the National Institutes of General Medical Sciences; The Johns Hopkins Medical School DHS Center on Preparedness and Catastrophic Event Response (PACER), under Award Number N00014-06-1-0991 from the Office of Naval Research; and The University of Pittsburgh Public Health Adaptive Systems (PHASYS) project, under Cooperative Agreement Number 1P01TP000304-01 from the Centers for Disease Control and Prevention.  Epstein's work is also supported by an NIH Director's Pioneer Award, Number DP1OD003874 from the Office of the Director, National Institutes of Health.  The content is solely the responsibility of the authors and does not necessarily represent the official views of the National Institute of General Medical Sciences, the National Institutes of Health, the Centers for Disease Control and Prevention, the Office of Naval Research, or the Office of the Director, National Institutes of Health. 

## Competing Interests

The authors have declared that no competing interests exist.

## Figures

**FIGURE 1**

**Figure:**
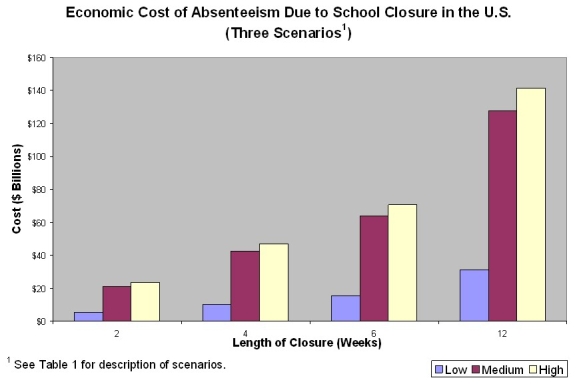

###                  

##  Tables

**TABLE 1**

**Table:** 

**Economic Costs of Absenteeism Due to School Closure in the United States (Billions of 2008 US dollars and Percent of 2008 GDP)**			
**Closure Length**	**Low Cost Estimate^1^**	**Base Estimate^2^**	**High Cost Estimate^3^**
2 Weeks	$5.2 (<0.1%)	$21.3 (0.1%)	$23.6 (0.2%)
4 Weeks	$10.6 (0.1%)	$42.6 (0.3%)	$47.1 (0.3%)
6 Weeks	$15.6 (0.1%)	$63.9 (0.4%)	$70.7 (0.5%)
12 Weeks	$31.3 (0.2%)	$127.8 (0.9%)	$141.3 (1.0%)

Sources: 2007 and 2008 CPS Outgoing Rotation Groups; 2008 CPS March Supplement; Child Care Module of the 2004 SIPP; Sadique et. al.; Harvard School of Public Health Project on the Public and Biological Security’s Pandemic Influenza Survey. 

^1^ Allows for use of informal care and work‑from‑home and assumes the elasticity of output with respect to hours worked is 0.8.  If a male and female are equally closely related to a child, the female misses work. ^2^ Assumes that an adult must miss work in each household with at least one child and the elasticity of output with respect to hours worked is 1.  If a male and female are equally closely related to a child, the female misses work. ^3^ Assumes that an adult must miss work in each household with at least one child and the elasticity of output with respect to hours worked is 1.  Assumes that households randomly choose whether males or females care for children.

**TABLE 2**

**Table:** 

**Weekly Cost Per Student^1^ (2008 US Dollars) **		
**Low Cost Estimate^2^**	**Base Estimate^3^**	**High Cost Estimate^4^**
$35	$142	$157
Sources: 2007 and 2008 CPS Outgoing Rotation Groups; 2008 CPS March Supplement; Child Care Module of the 2004 SIPP; Sadique et. al.; Harvard School of Public Health Project on the Public and Biological Security’s Pandemic Influenza Survey. ^1^ Cost per student is calculated as the total cost of absenteeism in the United States divided by the number of persons under 16 years of age or currently in high school. ^2^ Allows for use of informal care and work‑from‑home and assumes the elasticity of output with respect to hours worked is 0.8. If a male and female are equally closely related to a child, the female misses work. ^3^ Assumes that an adult must miss work in each household with at least one child and the elasticity of output with respect to hours worked is 1. If a male and female are equally closely related to a child, the female misses work. ^4^ Assumes that an adult must miss work in each household with at least one child and the elasticity of output with respect to hours worked is 1. Assumes that households randomly choose whether males or females care for children.		

## Endnotes

1

 These other industries include offices of health practitioners other than dentists, chiropractors, and optometrists, outpatient care centers, home health care services, hospitals, nursing care facilities, and other health care services. 2 We define relevant occupations liberally to include pharmacists, physicians and surgeons, registered nurses, health diagnosing and treating practitioners, clinical laboratory technologists and technicians, diagnostic related technologists and technicians, EMTs and paramedics, health diagnosing and treating practitioner support techs, licensed practical and licensed vocational nurses, medical records and health information technicians, miscellaneous health technologists and technicians, nursing, psychiatric and home health aides, medical assistants and other healthcare support occupations, and other healthcare practitioners and technical occupations.  We exclude chiropractors, dentists, dietitians and nutritionists, optometrists, podiatrists, occupational, physical, radiation, recreational, respiratory, and other therapists, speech-language pathologists, veterinarians, dental hygienists, opticians (dispensing), therapists’ and dentists’ assistants, and administrative workers. 3 Taking workers’ personal leave benefits into account would be an especially important contribution.  Persons with more leave might be more likely to stay home to care for children.  Once workers’ annual leave begins to run out, children may become more likely to resort to self-care, introducing important non-linearities into the relationship between the cost and length of school closure.  And workers’ absences may substitute for leave that would have been taken later in the year, instead of reducing annual output. 4 According to the Bureau of Labor Statistics’ Employer Costs for Employee Compensation data, benefits make up 30% of all compensation. 5 See CDC (2007), pp. 9-10 for definition of categories [1]. 6 We include all persons under 16 years of age or in high school as students for this calculation.  7 Of course our estimates are based on national demography data and do not take into account regional variation which might be salient at the local level. 8 Some may argue that household income quintiles are a poor measure of financial security because they do not account for household’s income needs, which vary by household size.  We recalculate income quintiles using household income adjusted for household size by dividing each household’s income by the square root of the number of persons in the household.  The impact remains disparate.  After this adjustment, a somewhat higher percentage of households with all of their income at risk belong to the second or fourth income quintile and fewer belong to the third. 9 The elasticity of output with respect to hours of labor is defined as the percent change in output that is brought about by a 1% change in hours worked. 10 Some of these household members would have stayed home anyway due to fear, the need to care for sick children, or their own illness.  Thus, our cost estimates are gross and not net.  A calculation of net cost would need to marry this economic analysis to a full blown epidemic model.  We save this project for future research. 11 This method differs from the method used by Sadique, Adams, and Edmunds (2008) because it assumes that the most common caretakers are children’s parents and family members, not necessarily the household’s head.  The estimated cost of school closure is similar if Sadique et. al.’s method for identifying caretakers is used. 12 Because we have no data on earnings of members of the Armed Forces, we assume that they are never available to care for their children in all of our estimates.  We believe this is a reasonable assumption.  In households where all adults are employed in the Armed Forces, we assume that children are not cared for by household members in the event of an epidemic.  13 Sadique et. al. first proposed this sensitivity test. 14 This approach assumes that all adults work at overlapping times.  It is possible that the aunt and uncle do not work at the same time and that no work need be missed in this household.  However, we know of no large public datasets that include the exact hours of the day all adults in a household are at work. 15 Family day care provision is typically in the home of a neighborhood adult and is much less formal than day care programs.  It therefore might still be available if schools and day care programs were closed during a pandemic. 16 An arrangement must be used at least once a week to be considered a “regular” arrangement. 17 This may slightly underestimate the availability of informal child care because some kids are cared for by parents who do not work and are not a part of their household.  Presumably, it would be easy for these children to be cared for without anyone in their household missing work.  Unfortunately, these children cannot be distinguished in the data. 18 This is a conservative assumption because some persons who provide child care in normal times work when they are not providing care.  Moreover, some child care providers provide care for more than one child, so not all households could expand their use of child care simultaneously without having many children cared for in the same room. 19 The Census does not report data on child care of 15 year olds, so we assume that they have informal child care available to them at the same rate as 0-14 year olds. 20 Because the survey was fielded in 2006, we use the CPI-U index to adjust the income groups for inflation. 21 The survey breaks out the ability to work from home for one month by household income level.  It also reports that 87% of those who could work from home and have major responsibility for household children would be able to care for those children while working from home.  To get the percent who could provide care while working from home, we multiply the percent who could work from home in each income category by 0.87. 22 CPS respondents report family income as a range.  To combine family incomes into household income, we assign income equal to the midpoint of the reported range.  Persons who refused to answer the income question are randomly assigned to an income group. 23 In the real world, not all industries are perfectly competitive and a worker’s compensation is not always equal to the value of her production.  Goods’ prices also do not always match their social value, so the total revenue lost due to absenteeism is not an exact measure of the economic cost of this absenteeism.  Therefore, estimates of economic cost will always be inexact. 24 This makes our cost estimate very conservative. 25 To impute these earnings, we first calculate average weekly earnings for each worker in 2007 by dividing annual earnings by weeks worked in the 2008 CPS ASEC.  We then take the mean of these earnings among self-employed male workers in households with kids and where every adult worked for at least one week in 2007.  In calculating these means, we multiply workers’ weights by the percent of weeks worked in 2007, inflating the weights of workers who were more likely to be in the labor force during any given week.  Call the result ERNSEM07.  We now have an estimate of weekly earnings among the self-employed in 2007 in addition to our main dataset with earnings in 2008.  We need to account for inflation and wage growth between 2007 and 2008.  Using the ORGs, we calculate the percent change in mean nominal weekly earnings from 2007 to 2008 among all male workers in households with kids and no stay-at-home adults.  Call this change DERNM.  We assume that earnings among male self-employed workers grew at the same rate as earnings among other male workers and assign ERNSEM07(1+DERNM) as the weekly earnings for all self-employed male workers in our main dataset.  We then repeat for female workers. 26 Sadique et. al. adjust for the presence of grandparents, but do not explain how they make this adjustment.  They do not adjust for the presence of other non-employed adults. 
